# Clinical and Biological Relevance of Kidney Injury Molecule-1 and Beta-2 Microglobulin in Monitoring Patients with Systemic Lupus Erythematosus

**DOI:** 10.3390/medicina61091663

**Published:** 2025-09-13

**Authors:** Corina Daniela Ene, Cristina Capusa, Ilinca Nicolae, Simona Roxana Georgescu, Cristina Iulia Mitran, Madalina Irina Mitran, Gheorghe Nicolae, Mircea Tampa, Clara Matei

**Affiliations:** 1 Department of Nephrology, ‘Carol Davila’ University of Medicine and Pharmacy, 020021 Bucharest, Romania; corina.ene@umfcd.ro (C.D.E.); cristina.capusa@umfcd.ro (C.C.); 2 Department of Nephrology, ‘Carol Davila’ Nephrology Hospital, 010731 Bucharest, Romania; 3 Research Department, ‘Victor Babes’ Clinical Hospital for Infectious Diseases, 030303 Bucharest, Romania; 4 Department of Dermatology, ‘Carol Davila’ University of Medicine and Pharmacy, 020021 Bucharest, Romania; dermatology.mt@gmail.com (M.T.); matei_clara@yahoo.com (C.M.); 5 Department of Dermatology, ‘Victor Babes’ Clinical Hospital for Infectious Diseases, 030303 Bucharest, Romania; 6 Department of Microbiology, ‘Carol Davila’ University of Medicine and Pharmacy, 020021 Bucharest, Romania; cristina.iulia.mitran@gmail.com (C.I.M.); madalina.irina.mitran@gmail.com (M.I.M.); 7 Faculty of Psychology, Babeș-Bolyai University, 400347 Cluj-Napoca, Romania; nicolaengheorghe@gmail.com

**Keywords:** systemic lupus erythematosus, renal injury molecule-1, beta2 microglobulin, kidney damage, skin damage

## Abstract

*Introduction:* Variations in kidney injury molecule-1 (KIM-1) and beta2-microglobulin (β2MG) levels, both involved in the pathogenesis of systemic autoimmunity, have been linked to tubulointerstitial lesions in patients with systemic lupus erythematosus (SLE). However, the significance of KIM-1 and β2MG in the pathogenesis and development of extrarenal manifestations in SLE remains unclear. This study aims to investigate the relationship between KIM-1 and β2MG levels, measured in both serum and urine, and their association with the clinical and biological features of SLE. *Materials and Methods:* KIM-1 and β2MG levels were measured in 80 adult patients with SLE (who exhibited mucocutaneous, hematological, and renal manifestations) and 30 control subjects. All patients with renal abnormalities related to SLE underwent a renal biopsy. The serum and urinary levels of KIM-1 (measured in pg/mL for serum and ng/mL for urine) and β2MG (measured in ng/dl for serum and mg/l for urine) were determined for each subject using the ELISA method and immunoturbidimetry, respectively. *Results:* There were significant differences in the serum and urinary levels of KIM-1 and β2MG between the SLE group and the control group, as well as among subgroups with different manifestations (renal, cutaneous, and hematological). Elevated levels of KIM-1 and β2MG, in both serum and urine, were associated with the clinical activity of the disease, the inflammatory process, and the development of tissue damage in various organs, leading to declines in renal function, hematological disorders, and mucocutaneous manifestations. *Conclusions:* KIM-1 may play a pathogenic role in kidney injury and disease, while β2MG could have a pathogenic role in both kidney and non-kidney diseases. In summary, KIM-1 characterizes renal involvement, while serum β2MG correlates with the progression of cumulative lesions in SLE patients. Our findings could enhance early diagnosis, predict disease progression, and elucidate the pathogenic mechanisms underlying SLE.

## 1. Introduction

Systemic lupus erythematosus (SLE) is an autoimmune disease that affects various systems and is characterized by a complex pathophysiology and variable clinical manifestations. The development of SLE is the result of intricate interactions among sociodemographic, genetic, epigenetic, molecular, immunological, and hormonal factors. These interactions trigger the production of autoantibodies that cause tissue damage in various organs due to the activation of numerous pro-inflammatory pathways [1,2,3].

A common and serious complication of SLE that necessitates careful evaluation is kidney damage. Renal involvement in SLE is an immunoglobulin complex disease. Early detection of signs of renal failure in patients with SLE through appropriate diagnostic tests, such as renal biopsy and imaging, aims to maintain renal function and stop evolution to end-stage renal disease [4,5,6,7,8]. The excessive activation of B and T cells, the production of autoantibodies, and the imbalance between the formation and clearance of immune complexes, along with the interactions between infiltrating immune cells and resident kidney cells, significantly impact renal pathology and extrarenal manifestations in SLE [3,9,10].

Among the various organs affected by SLE, the skin is the second most commonly involved, with about 80% of patients experiencing skin manifestations during the disease progression [1]. Skin involvement is a frequent comorbidity in SLE patients that is associated with kidney disease in a neutrophil-dependent manner, indicating a connection between kidney and skin health [1,8,11]. Research shows a direct link between skin inflammation and kidney damage. It has been demonstrated that neutrophils migrate from the blood to skin that has been exposed to UV rays and subsequently spreads throughout the body. These circulating immune cells interact with various kidney cell types, including glomerular epithelial cells, mesangial cells, podocytes, and tubular epithelial cells, thereby initiating autoimmunity. In the kidneys, neutrophils mediate inflammatory responses, supporting the hypothesis that skin lesions could contribute to kidney damage in SLE patients [11]. Additionally, studies indicate that 5% to 25% of patients with cutaneous lupus erythematosus, who show increased levels of antinuclear antibodies (ANA), anti-double-stranded DNA (anti-dsDNA) antibodies, and anti-Smith (anti-SM) antibodies, may progress to SLE [1].

Identifying specific biomarkers for early diagnosis, prognosis, monitoring, or treatment in patients with SLE can be a challenging task. Recent reports indicate that renal injury molecule-1 (KIM-1) and Beta-2 microglobulin (β2MG), both involved in the development of systemic autoimmunity, may play a significant role in managing SLE patients, who are at a higher risk of tissue damage in multiple organs. The correlation of KIM-1 and β2MG with the clinical activity of SLE, along with their detectable levels in urine and plasma, underscores the potential clinical utility of these biological factors [12,13,14,15,16].

KIM-1/TIM-1 is a type I transmembrane glycoprotein expressed in various tissues, and its expression is rapidly induced at both the gene and protein levels. In healthy kidneys, KIM-1 expression is low, but it becomes significantly elevated in renal lesions of various etiologies [17]. KIM-1 is upregulated in patients with proximal tubular renal injury [13,17]. The activity of KIM-1 is associated with physiological processes and is closely linked to the adaptive responses of renal epithelium to ischemic or toxic lesions, as well as various pathophysiological conditions such as acute kidney injury, chronic kidney disease, acute and chronic graft rejection, nephrotoxicity, ischemia, dehydration, inflammation, malignant tumors, and the progression of fibrosis [13,17]. KIM-1 is primarily secreted in the kidneys. Its extracellular domain is released into the tubular lumen through a metalloproteinase-mediated process, making it detectable in both urine and blood [12,18]. Additionally, KIM-1 plays crucial roles in regulating inflammation and modulating immune responses [12,19].

β2MG is a low molecular-weight protein that shares sequence homology with immunoglobulins. It acts as a component of the major histocompatibility complex class I (MHC-I) and has been recognized as a regulator of inflammation, as well as a potential modulator of the immune response and systemic autoimmunity [20,21,22]. Under normal physiological conditions, β2MG is continuously produced by lymphocytes, catabolized by renal proximal tubular cells, and cleared from circulation via the kidneys. It exists in two forms: membrane-bound β2MG and soluble free β2MG [23]. The soluble form is upregulated in patients with autoimmune diseases, lymphoproliferative disorders, renal glomerular disorders, neutrophilic inflammatory skin disorders, skin cancer, and oral neoplasms [10,24]. In lupus nephritis, β2MG levels are thought to reflect disease activity [1,9,25,26]. Recent studies show that free β2MG can promote cell growth, migration, programmed cell death, and metastasis, making it a key prognostic indicator and predictor of survival in different types of skin malignancies [23].

Currently, there is limited data on the relationship between KIM-1 and β2MG, along with the clinical and biological characteristics of patients with SLE. This study aims to evaluate the potential relationship between serum and urinary levels of KIM-1 and β2MG and (a) disease activity, and (b) the development of tissue lesions (including renal damage, hematological abnormalities, and skin lesions) in SLE patients.

## 2. Materials and Methods

### 2.1. Study Design

The present study is a case-control study performed between 2021 and 2025 in the Clinical Hospital of Nephrology, “Dr Carol Davila”, Bucharest, Romania. The subjects included in the study were divided into two groups: the SLE group that included 80 SLE patients with hematological, cutaneous–mucosal, and renal determinations and the control group with 30 healthy subjects. All the subjects were included in the study, after signing the informed consent statement; the Declaration of Helsinki from 1975 was respected. The study protocol was approved by the Ethics Committee of the Clinical Hospital of Nephrology, “Carol Davila” (26/25 October 2021).

SLE diagnosis was based on immunological determinations and renal biopsy, according to Systemic Lupus International Collaborating Clinics/American College of Rheumatology criteria. All the subjects with active urinary sediment underwent renal biopsy, with evaluation in optical and electronic microscopy and immunofluorescence, according to KDIGO guidelines. SLE disease activity was evaluated by the Systemic Lupus Erythematosus Disease Activity Index (SLEDAI). The study included SLE subjects who had chronic kidney disease at stages 1 or 2, with an estimated glomerular filtration rate (eGFR) greater than 60 mL/min/1.73 m^2^. All participants had stable disease and were receiving treatment, which included non-steroidal anti-inflammatory drugs, corticosteroids, hydroxychloroquine, immunosuppressant medications (such as azathioprine and mycophenolate mofetil), and antihypertensive therapy, for at least six months. Information about the ongoing treatment for each patient was recorded.

The exclusion criteria comprised the following: individuals under 18 years of age, pregnant women, tobacco users, those taking vitamins or antioxidants, and patients with stage 3 to 5 chronic kidney disease. Additionally, individuals with any cardiovascular, hepatic, thyroid, gastrointestinal, or oncological diseases, as well as those who had experienced any viral or bacterial infections in the previous three months, were excluded from the study.

### 2.2. Clinical and Biological Characteristics of the Studied Groups

The SLE group included 46 women with a mean age of 45 ± 12 years and 34 men with a mean age of 49 ± 17 years. The control group included 15 women with a mean age of 41 ± 9 years and 15 men with a mean age of 44 ± 21 years. The SLE group comprised 48 subjects presenting with cutaneous and hematological manifestations but without lupus nephritis, and 32 subjects diagnosed with lupus nephritis. The classic biomarkers of lupus activity that were evaluated were antinuclear antibodies, anti dsDNA antibodies, urine albumin-to-creatinine ratio (uACR), and complement components C3 and C4. dsDNA had significantly high values in subjects with lupus nephritis compared with SLE subjects with hematological and cutaneous–mucosal determination and the control group. The complement proteins had high levels in SLE groups compared to the control group. Leucocytes and hemoglobin were statistically significantly lower in SLE groups compared with the control group.

### 2.3. Laboratory Tests

After signing the informed consent statement, each subject included in the study underwent blood sampling after a 12 h fasting period, using a holder–vacutainer system and the urine samples were collected from the first urine in the morning. We determined the serum and urinary levels of KIM by ELISA method (Serum and urinary KIM-1-Reactivity-Human, ELISA kit-Elabscience, Catalog number-E-EL-H6029; TECAN analyzer-GmbH, Grodig, Austria). Serum β2MG was quantified from peripheral venous blood collected a jeun, along with urinary β2MG, using an immunoturbidimetric assay that employed a latex-bound rabbit anti-β2MG polyclonal antibody. The Beta-2 Microglobulin Protein Kit, Human (HEK293, His), and HumaStar300 analyzer (Weisbaden, Germany) were utilized.

### 2.4. Statistical Analysis

The data are presented as means and standard deviations. The comparison tests employed include Analysis of Variance (ANOVA) with Tukey’s post hoc test, or the Kruskal–Wallis test with Dunn’s post hoc test, depending on whether the data are normally or non-normally distributed. To assess the relationship between the studied parameters (KIM-1 and β2MG) and eGFR, SLEDAI, and various clinical features of SLE, Pearson’s correlation coefficient was used. Prior to evaluating these relationships, data normality was assessed using the Kolmogorov–Smirnov test. A significance level (*p*-value) of 0.05 (5%) was chosen, and a confidence interval of 95% was used.

## 3. Results

Serum and urinary levels of KIM-1 (ng/dL serum, ng/mL urine) and β2MG (ng/dL serum, mg/L urine) were significantly different between SLE patients and controls (Table 1).

The serum and urinary values of KIM-1 (ng/dL serum, ng/mL urine) and β2MG (ng/dL serum, mg/L urine) in patients with SLE were significantly different according to the values of eGFR (Table 2), albuminuria (Table 3), and active urinary sediment (hematuria, pyuria, cylindruria).

The KIM-1 and β2MG variations in patients with SLE were inconsistently affected by SLEDAI levels. Serum β2MG levels were significantly associated with SLE activity (Table 4).

sKIM-1 and uKIM-1, in patients with SLE, were not influenced by the presence of skin determinations. It is interesting to note that sβ2MG levels vary significantly in patients with mucocutaneous findings (ulcers, lesions of the oral mucosa, and alopecia) (Table 5).

KIM-1 and β2MG levels in patients with SLE vary moderately in the presence of hematological disorders, such as anemia, leukopenia, and thrombocytopenia (Table 6). Additionally, KIM-1 and β2MG levels in patients with SLE are significantly impacted by chronic inflammation, as indicated by markers such as erythrocyte sedimentation rate (ESR) and α1-acid glycoprotein (AGP) (Table 6).

An analysis of the relationships between serum and urinary levels of KIM-1 and β2MG, as well as clinical features and biological data of SLE patients, is summarized in Table 6. Urinary KIM-1 levels show a strong positive association with increased levels of albuminuria, active urinary sediment (including hematuria, leukocyturia, and cylindruria), and inflammatory factors. In contrast, urinary KIM-1 levels are inversely correlated with eGFR values.

Serum KIM-1 levels are also strongly positively correlated with high levels of albuminuria and are independent of eGFR and SLEDAI. A positive correlation was found between serum and urinary KIM-1 values.

**Figure 1:**
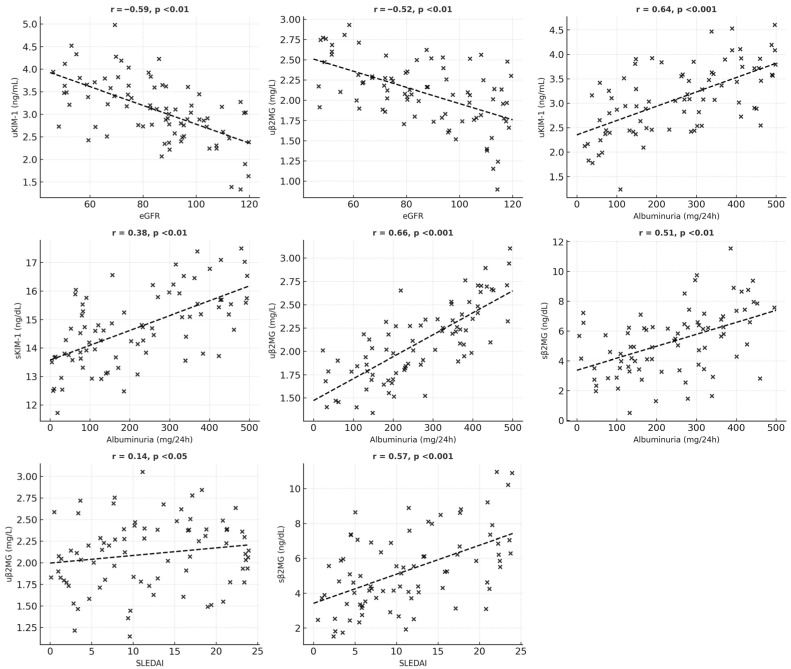
Schematic representation of significant correlations between the studied parameters.

**Figure 2:**
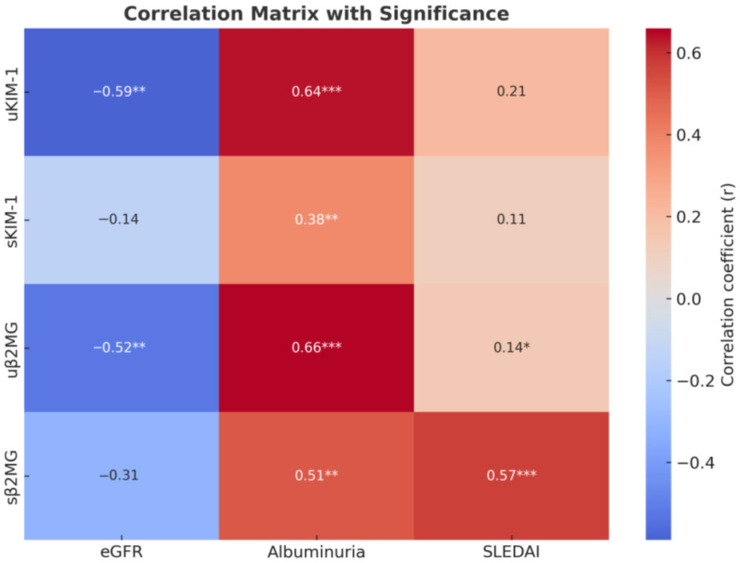
Correlation heatmap. * *p* < 0.05, ** *p* < 0.01, and *** *p* < 0.001.

For β2MG, both serum and urinary levels were associated with increased albuminuria and markers of inflammation; however, only urinary β2MG correlated with lower eGFR and active urinary sediment. Serum β2MG levels show a significant positive correlation with SLEDAI scores and the presence of mucocutaneous manifestations (such as ulcers, lesions of the oral mucosa, and alopecia) and vary moderately with hematological abnormalities (including anemia, leukopenia, and thrombocytopenia). A statistically significant positive correlation was also identified between serum and urinary β2MG levels (Figure 1 and Figure 2).

## 4. Discussion

The findings from this study reinforce the role of KIM-1 and β2MG as important markers in the early diagnosis, disease progression, and understanding of the pathogenic basis of SLE. Through a systematic quantitative evaluation of KIM-1 and β2MG levels in both urine and blood samples, we compared a representative group of SLE patients with control subjects and obtained the following conclusions (see Table 7):

(a) The elevated levels of KIM-1 and β2MG in patients with SLE compared to controls suggest that these biomarkers play a significant role in the pathophysiology of SLE. The measurement of KIM-1 and β2MG could serve as a molecular prognostic model for individuals diagnosed with SLE.
(b) Both serum and urinary levels of KIM-1 and β2MG are associated with the clinical and biological manifestations of SLE.
(c) KIM-1 is a marker that defines the renal phenotype in SLE patients.
(d) β2MG reflects the progression of cumulative lesions in individuals with SLE.
(e) Serum β2MG levels can distinguish between patients with renal lesions and those without renal lesions.

Our findings align with other published research regarding the pathophysiological roles of KIM-1 and β2MG in patients with SLE. By comparing the urinary and serum levels of these two parameters with those of a control group, we have demonstrated the significance of KIM-1 and β2MG in SLE pathogenesis. Variations in the levels of KIM-1 and β2MG in SLE patients correlate with the severity of the disease, the presence of tissue lesions (including renal, hematological, and cutaneous lesions), and the extent of inflammation.

Several studies highlight the pathophysiological role of KIM-1 in various kidney disorders. Significantly higher levels of KIM-1 have been detected in patients with acute ischemia, toxic kidney injury, renal cell carcinoma, chronic kidney disease, and lupus nephritis [19]. Urinary KIM-1 levels are associated with disease activity and histological lesions in lupus nephritis [19,32]. Therefore, KIM-1 may serve as a valuable biomarker for monitoring SLE. The value of this biomarker lies in its detectability in various biological samples (urine, blood, and both normal and pathological tissue), its potential to identify lupus nephritis at an early stage, and its ability to monitor the progression of kidney disease.

The upregulation of KIM-1 in SLE may be attributed to accelerated proteolytic cleavage of the KIM-1 ectodomain in renal lesions [2,29]. KIM-1 plays a role in regulating cell-to-cell adhesion and endocytosis [33]. The ectodomain of KIM-1 undergoes proteolytic cleavage, mediated by ADAMs 10 and 1, as well as MMPs 3 and 14, early in the lesion formation, leading to its release into the blood and urine [28,29]. Although KIM-1 is overexpressed in renal cell carcinoma, the plasma levels of KIM-1 do not correlate with the severity of kidney damage in metastatic renal cancer, suggesting that circulating KIM-1 mainly originates from the tumor [13,34]. Thus, plasma KIM-1 measurement can help identify early-stage renal cancer and monitor disease progression [17]. This data supports KIM-1 value as a novel biomarker for kidney injury and disease.

Consistent with a significant body of the published literature, our results indicate that β2-MG levels are considerably higher in SLE patients than in controls. The levels of β2-MG progressively increase with the development of lesions in multiple organs and tissues. A recent meta-analysis that included 16 studies with 1368 SLE patients and 423 controls revealed persistent overexpression of β2-MG in SLE patients. The analysis found a strong correlation between β2-MG levels and disease activity and noted a significant reduction in β2-MG levels in patients receiving immunosuppressive treatment [10]. An experimental study conducted on mice with SLE and β2-MG deficiency indicated differences in the clinical presentation of the disease [15]. The increase in β2-MG in SLE may result from high lymphocyte turnover typically seen in autoimmune diseases, aberrant activation of B and T cells, overproduction of autoantibodies targeting β2-MG, and the kidney’s inability to filter excessive immune complexes [10,15].

In recent years, numerous studies have highlighted the significant biological roles of β2MG in tumor immunity. Research suggests that a deficiency in the β2MG gene may inhibit the presentation of MHC class I molecules, thereby promoting immune evasion in melanoma [23]. In squamous cell carcinoma of the oral cavity, the expression of β2MG has been positively correlated with cell migration and invasion. Furthermore, intense immunohistochemical staining of β2MG serves as an indicator of reduced 5-year survival rates in oral cancer patients and is associated with advanced tumor stages and positive lymph node status [24]. Notably, β2MG is found in the plasma membrane of normal oral mucosa cells, but translocates into the cytoplasm in oral squamous cell carcinoma. These findings suggest that the mechanism of β2MG-mediated tumorigenesis involves the internalization of the protein from the cell surface into the cytoplasm of tumor cells. β2MG functions as a pleiotropic signaling molecule, modulating the tumor immune microenvironment through a ligand–receptor interaction mechanism. Monoclonal antibodies against β2MG can induce apoptosis in malignant cells. Therefore, β2MG is considered an important prognostic and survival factor in various malignancies [23].

Additionally, this research has provided valuable insights for nephrology and dermatology, particularly regarding the potential pathogenic role of KIM-1 in kidney injury and disease, and has also highlighted the relevance of β2MG in chronic lesions in patients with SLE. KIM-1 levels show a relationship with eGFR values and the pathological characteristics of urinary sediment, such as leukocyturia, hematuria, and cylindruria. This relationship is attributed to the ability of KIM-1 to regulate the adaptive responses of the renal epithelium to ischemic lesions and toxins [30].

Our study also indicated that β2MG is correlated with mucocutaneous damage in SLE. Circulating levels of β2MG were significantly higher in SLE patients with cutaneous and mucosal lesions (such as alopecia and oral ulcers) compared to those without skin involvement. Serum β2MG levels can distinguish between SLE patients with and without skin manifestations. An experimental study on mice with SLE and high levels of β2MG revealed an increased number of mice exhibiting skin symptoms, while fewer showed kidney damage [15]. Other research has indicated that elevated serum β2MG levels are associated with conditions such as serositis, oral ulcers, and lupus nephritis [10,25]. Moreover, serum β2MG levels rise in SLE patients with arthritis, skin symptoms, and/or mucosal involvement, lupus nephritis, and cardiac disorders, but not in those with hematologic, neurological, or ocular abnormalities [29]. The increase in serum β2MG levels among SLE patients with skin pathology may stem from a disruption in the interaction between β2MG and MHC class I/HLA-I. Typically, β2MG orchestrates MHC class I/II expression, the production of immunosuppressive molecules, interferon-gamma (IFN-γ) secretion, T cell autoreactivity, and the regulatory mechanisms of immune responses [16,29,35]. Disruption of the β2MG and MHC class I/HLA-I interaction adversely affects mucosal immunity [20].

In autoimmune disorders, the role of lipid rafts in LRP signaling, which is triggered by autoantibodies in endothelial cells, has been discussed. In SLE, autoantibodies induce the expression of tissue factors in endothelial cells through the LRP signal transduction pathway. This autoantibody-mediated signaling is facilitated by lipid rafts, which are microdomains of the plasma membrane enriched in glycosphingolipids and cholesterol. Similar lipid raft-dependent processes may also be significant for the physiological role of β2MG in cell signaling and vascular pathogenesis. The results of our study provide new insights into the pathogenesis of SLE [36].

Recent research demonstrates a positive relationship between serum β2MG levels and the cytokines involved in the pathogenesis of SLE, such as IL-6, IL-8, IL-18, and IFN-α [10]. Furthermore, β2MG promotes the polarization of M1 macrophages while inhibiting M2 polarization. It also induces the expression of cytokines and mediates their interaction with receptors [31].

KIM-1 regulates the inflammatory response by interacting with p85, PI3K, NF-κB, and STAT3/ERK1/2. It facilitates the phagocytosis of apoptotic cells, thereby protecting the kidney by acting as an inhibitor of innate immune response and inflammation [27,28].

The evaluation of the biomarkers KIM-1 and β2MG, both in serum and urine, is a significant aspect of the study. The findings indicate that KIM-1 is primarily specific to the kidneys, while β2MG reflects both renal and systemic diseases. These biomarkers can be detected through non-invasive tests using urine and serum, and they may indicate changes at the cellular or molecular level. Although β2MG and KIM-1 are not sufficient for diagnosing SLE, they can be valuable in suggesting the presence, development, and progression of this systemic disease. Ultimately, additional studies are needed to explore the relationship between variations in these biomarkers and the response to treatment.

By rigorously monitoring these two parameters, future research should validate the potential of these non-invasive biological factors for use in the diagnosis, clinical prognosis, and guidance of therapeutic strategies in patients with SLE.

## 5. Conclusions

This study provides compelling evidence that, in patients diagnosed with SLE, KIM-1 may play a role in kidney injury and disease, while β2MG could be involved in both kidney and non-kidney diseases. In conclusion, these biomarkers correlate with organ involvement and disease activity; however, their mechanistic contribution remains to be established.

## Figures and Tables

**Table 1:** Serum and urinary levels of KIM-1 and β2MG in SLE patients and controls.

Variable	SLE Patients (*n* = 80)	Controls (*n* = 30)	*p*-Value
uKIM-1 (ng/mL)	3.1 ± 0.7	1.4 ± 0.3	<0.01
sKIM-1 (ng/dL)	14.7 ± 1.3	8.8 ± 0.5	<0.001
uβ2MG (mg/L)	2.1 ± 0.4	0.1 ± 0.1	<0.001
sβ2MG (ng/dL)	5.3 ± 2.2	1.3 ± 0.4	<0.001

Data are presented as mean ± standard deviation; uKIM-1: urinary Kidney Injury Molecule-1; sKIM-1: serum Kidney Injury Molecule-1; uβ2MG: urinary β2-microglobulin; and sβ2MG: serum β2-microglobulin.

**Table 2:** Serum and urinary levels of KIM-1 and β2MG in SLE, according to eGFR.

Variable	eGFR ≥90 (A) (*n* = 31)	eGFR 75–90 (B) (*n* = 29)	eGFR 60–75 (C) (*n* = 20)
uKIM-1 (ng/mL)	2.2 ± 0.3	2.7 ± 0.5	4.7 ± 1.2
sKIM-1 (ng/dL)	9.2 ± 0.5	15.5 ± 1.0 *	22.7 ± 1.5
uβ2MG (mg/L)	0.3 ± 0.1	2.2 ± 0.4 *	4.8 ± 1.1
sβ2MG (ng/dL)	3.4 ± 0.9	4.9 ± 1.1 *	8.7 ± 1.8

Data are presented as mean ± standard deviation; * *p* < 0.05 statistical significance B versus A, and C versus A; eGFR: estimated Glomerular Filtration Rate; uKIM-1: urinary Kidney Injury Molecule-1; sKIM-1: serum Kidney Injury Molecule-1; uβ2MG: urinary β2-microglobulin; and sβ2MG: serum β2-microglobulin.

**Table 3:** Serum and urinary levels of KIM-1 and β2MG in SLE, according to albuminuria level.

Parameter	Albuminuria < 30 mg/24 h (A)	Albuminuria 30–299 mg/24 h (B)	Albuminuria 300–500 mg/24 h (C)
No of patients	33	31	16
uKIM-1 (ng/mL)	2.1 ± 0.3	3.5 ± 0.5	4.5 ± 1.1
sKIM-1 (ng/dL)	9.8 ± 0.4	17.7 ± 1.1 *	20.2 ± 1.3
uβ2MG (mg/L)	0.2 ± 0.1	2.9 ± 0.6 *	4.4 ± 1.3 */**
sβ2MG (ng/dL)	2.4 ± 0.3	5.9 ± 1.0 *	9.9 ± 2.0 */**

Data are presented as mean ± standard deviation. * *p* < 0.05 statistical significance B versus A and C versus B; ** *p* < 0.010 statistical significance C versus A; uKIM-1: urinary Kidney Injury Molecule-1; sKIM-1: serum Kidney Injury Molecule-1; uβ2MG: urinary β2-microglobulin; and sβ2MG: serum β2-microglobulin.

**Table 4:** Serum and urinary levels of KIM-1 and β2MG in SLE, according to SLEDAI.

Parameter	SLEDAI < 5 (A)	SLEDAI 5–11 (B)	SLEDAI ≥ 11 (C)
No of patients	14	49	17
uKIM-1 (ng/mL)	2.8 ± 0.4	2.9 ± 0.9	3.9 ± 1.1
sKIM-1 (ng/dL)	13.4 ± 1.1	13.1 ± 1.9 *	20.9 ± 2.2
uβ2MG (mg/L)	0.3 ± 0.2	2.5 ± −0.6 *	2.4 ± 0.4
sβ2MG (ng/dL)	3.9 ± 0.7	4.8 ± 1.3 *	7.7 ± 2.1

Data are presented as mean ± standard deviation. * *p* < 0.05 statistical significance B versus A and C versus A; uKIM-1: urinary Kidney Injury Molecule-1; sKIM-1: serum Kidney Injury Molecule-1; uβ2MG: urinary β2-microglobulin; and sβ2MG: serum β2-microglobulin, SLEADAI—systemic lupus erythematosus disease activity index.

**Table 5:** Serum and urinary levels of β2MG, depending on cutaneous manifestations, in patients with SLE at the same stage of evolution.

Variable	No. of Cases	uβ2MG (mg/L)	sβ2MG (ng/dL)
Alopecia	Present (18)	3.0 ± 0.6	5.4 ± 1.0
	Absent (11)	2.9 ± 0.8	4.9 ± 1.3
*p*		*p* > 0.05	*p* < 0.01
Oral ulcers	Present (14)	2.7 ± 0.6	4.0 ± 0.8
	Absent (22)	2.6 ± 0.8	3.4 ± 0.5
*p*		*p* > 0.05	*p* < 0.01

Data are presented as mean ± standard deviation. uβ2MG—urinary beta-2 microglobulin; and sβ2MG—serum beta-2 microglobulin.

**Table 6:** Correlations between β2MG, KIM-1, and the clinical/biological characteristics of SLE patients.

Variable	uKIM-1	sKIM-1	uβ2MG	sβ2MG
eGFR	**r = −0.59** ***p* < 0.01**	r = −0.144 *p* = 0.05	**r = −0.52** ***p* < 0.01**	r = −0.31 *p* > 0.05
Albuminuria	**r = 0.64** ***p* < 0.001**	**r = 0.38** ***p* < 0.01**	**r = 0.66** ***p* < 0.001**	**r = 0.51** ***p* < 0.01**
SLEDAI	r = 0.21 *p* = 0.05	r = 0.11 *p* > 0.05	r = 0.14 ***p* < 0.05**	r = 0.57 ***p* < 0.001**
Hematuria	r = 0.25 ***p* < 0.05**	r = 0.19 ***p* < 0.05**	r = 0.10 *p* > 0.05	r = 0.16 *p* > 0.05
Leukocyturia	r = 0.14 ***p* < 0.05**	r = 0.27 ***p* < 0.05**	r = 0.33 ***p* < 0.05**	r = 0.07 *p* > 0.05
Cylindruria	r = 0.165 *p* > 0.05	r = 0.102 *p* > 0.05	r = 0.13 *p* > 0.05	r = 0.17 *p* > 0.05
Hemoglobin	r = 0.04 *p* = 1.0	r = 0.08 *p* > 0.05	r = 0.18 *p* > 0.05	r = −0.12 *p* > 0.05
Leukocytes	r = −0.07 *p* = 0.97	r = 0.02 *p* > 0.05	r = −0.11 *p* > 0.05	r = −0.39 *p* < 0.05
Thrombocytes	r = 0.02 *p* = 1.0	r = −0.11 *p* > 0.05	r = 0.07 *p* > 0.05	r = 0.04 *p* > 0.05
Mucocutaneous manifestations	r = 0.056 *p* > 0.05	r = 0.133 *p* > 0.05	r = 0.038 *p* > 0.05	r = 0.491 ***p* < 0.001**
ESR	r = 0.401 ***p* < 0.001**	r = 0.278 ***p* < 0.010**	r = 0.211 ***p* < 0.010**	r = 0.364 ***p* < 0.010**
AGP	r = 0.398 ***p* < 0.010**	r = 0.103 ***p* < 0.05**	r = 0.277 ***p* < 0.010**	r = 0.201 ***p* < 0.010**
sKIM	r = 0.58 ***p* < 0.05**	-	r = 0.129 ***p* < 0.05**	r = 0.146 *p* > 0.05
sβ2MG	r = 0.13 *p* = 0.05	-	r = 0.425 ***p* < 0.05**	-
uβ2MG	r = 0.216 ***p* < 0.05**	-	-	-

uKIM-1—urinary Kidney Injury Molecule-1; sKIM-1—serum Kidney Injury Molecule-1; uβ2MG -urinary Beta-2 microglobulin; sβ2MG—serum Beta-2 microglobulin; eGFR: estimated Glomerular Filtration Rate; SLEADAI score: systemic lupus erythematosus disease activity; ESR—erythrocyte sedimentation rate; and AGP—α1-acid glycoprotein. r = correlation coefficient.

**Table 7:** Signature of KIM-1 and β2MG in SLE progression.

Role of KIM-1 and β2MG in SLE	KIM-1 Profile	β2MG Profile	Potential Mechanisms
Pathogenesis	Serum levels of KIM-1 show significant increase in SLE (Table 1). KIM-1 could represent (a) a molecular factor for detecting lupus nephritis before clinical diagnosis; (b) a prognostic model for patients with SLE.	β2MG is elevated in the serum of patients with SLE. β2MG substantially improved discrimination between cases and controls.	The KIM-1 upregulation in SLE would be explained by the accelerated proteolytic cleavage of the KIM-1 ectodomain in the early stage of the renal disease [27,28]. Increased lymphocyte turnover could explain β2MG upregulation in SLE [10,15].
Skin and/or mucosal lesions	Invariable serum KIM-1 values in patients with SLE with preserved renal function and cutaneous determinations. KIM-1 could be an indicator of renal status in SLE.	Serum β2MG levels are high in patients with SLE and skin complications. β2MG has a potential pathogenic element in non-renal diseases.	β2MG and MHC-I/HLA-I orchestrate mucosal immunity, production of immunosuppressive molecules, and melanocyte stimulating hormone activity [16,20,29].
Development of renal manifestations	KIM-1 levels increase with worsening proteinuria, and decrease eGFR and abnormal urinary findings (leukocyturia, hematuria, and cylindruria).	β2MG levels increase progressively with decreasing eGFR and rising proteinuria.	KIM-1 mediates adaptive reactions of the renal epithelium to ischemic lesions [30]. The overproduction of β2MG favors the formation of immune complexes and impairs glomerular function [10].
Inflammatory profile	KIM-1 levels increase with serum pro-inflammatory factors (ESR, AGP) in SLE.	β2MG levels increase in inflammatory processes.	KIM-1 regulates NF κB, STAT3/ERK1/2. β-MG promotes M1/M2 polarization [28,31].
Disease activity	KIM-1 correlates with disease severity.	β2MG levels vary significantly with SLEDAI.	KIM-1 and β2MG levels vary with the clinical course of SLE [13,16].

## Data Availability

All data are contained within the article.
